# Exploring the Normalisation of Telepsychiatry Practice Among Private Psychiatrists in Australia: A Convergent Mixed Methods Study

**DOI:** 10.1155/ijta/4100418

**Published:** 2025-12-18

**Authors:** Luke Sy-Cherng Woon, Paul A. Maguire, Jeffrey C. L. Looi, Rebecca E. Reay

**Affiliations:** ^1^ Academic Unit of Psychiatry & Addiction Medicine, The Australian National University School of Medicine and Psychology, Canberra, Australia; ^2^ Department of Psychiatry, Faculty of Medicine, The National University of Malaysia, Kuala Lumpur, Malaysia, ukm.my; ^3^ Consortium of Australian-Academic Psychiatrists for Independent Policy Research and Analysis, Canberra, Australia

**Keywords:** implementation science, private practice, psychiatry, qualitative research, telehealth

## Abstract

**Objective:**

The widespread use of telepsychiatry requires a better understanding of its integration into routine practice. Using the normalisation process theory (NPT), we aimed to measure and explore Australian psychiatrists′ normalisation of telepsychiatry into private practice.

**Methods:**

This convergent parallel‐mixed methods study consists of an online survey of private psychiatrists using the Normalisation Measures Development (NoMAD) instrument and semistructured in‐depth interviews of private psychiatrists. We measured normalisation levels by the NPT constructs. We used logistic regression analyses to examine individual (psychiatrist) and practice factors for higher telepsychiatry patient load. We also thematically analysed the interviews according to the NPT. Finally, we integrated the quantitative and qualitative findings and drew inferences on factors for greater telepsychiatry integration.

**Results:**

We surveyed 110 respondents. Higher cognitive participation and collective action were associated with greater telepsychiatry use (> 50% patient load). The high‐load group was less likely to view telepsychiatry as different from usual work practice. Solo practices, and practices covering nonmetropolitan areas, were associated with a higher telepsychiatry load. Thirteen interviews were conducted. Telepsychiatry was largely regarded as equivalent, accessible, flexible and efficient. A conducive environment (resources, policy frameworks and general acceptance) promoted adoption. Telepsychiatry′s adverse effects on work quality and well‐being and its limitations and potential misuse were noted. In implementing telepsychiatry, perceived similarity to conventional consultations, bottom‐up initiatives tailored to individual needs, confidence and skills and personal appreciation of its benefits were important.

**Conclusions:**

Telepsychiatry is a useful tool that can be successfully integrated into routine private practice with adequate support.

## 1. Introduction

In Australia, Medicare‐reimbursed private practice telepsychiatry for rural and remote areas has existed since the early 2000s [1]. During the COVID‐19 pandemic, it became available nationwide and was widely used [2]. For better healthcare service planning, we need to understand how telepsychiatry, a relatively complex and new form of service delivery, is best implemented and integrated as part of routine clinical practice [3]. It is necessary to clarify how service providers (private practice psychiatrists) are involved in the implementation process.

Globally, various studies have examined clinicians′ experience in using telepsychiatry. Quantitative surveys, such as the 2023 survey of American Psychiatric Association members [4] and psychiatrist surveys in India [5, 6], indicated high usage and satisfaction and reported barriers, such as funding, and regulatory and technical issues. A recent multinational survey confirmed a high satisfaction and acceptance of telepsychiatry [7]. Several qualitative studies involved semistructured interviews or open‐ended questionnaires to explore clinicians′ views more in‐depth, discussing the advantages and challenges of telepsychiatry [8–10], as well as specific applications, such as use in consultation–liaison psychiatry [11] and developing culturally safe telepsychiatry [12]. A mixed methods study of British and Italian clinicians, patients and caregivers employed questionnaires and focus groups to explore differences in their perspectives on telepsychiatry [13].

Notably, few studies have specifically examined psychiatrists′ opinions on the implementation of telepsychiatry in practice. Implementing complex interventions in healthcare institutions involves more than the basic adoption and diffusion of innovations. In Australia, private psychiatrists provide a considerable portion of the outpatient psychiatric services, yet their experiences with telepsychiatry remain underresearched. Few previous Australian studies adopted qualitative or mixed methods approaches to study clinicians′ perspectives in telepsychiatry implementation [14]. Given the regulatory and reimbursement policy concerns of telepsychiatry [15], it is proper to investigate the implementation of telepsychiatry within the unique ecosystem of the Australian private psychiatry sector, particularly the Medicare item numbers that determine the financial viability of telepsychiatry services.

There is a relative paucity of theory‐based research into telepsychiatry implementation and usage in the Australian context and specifically since the government‐reimbursed COVID‐19 expansion of access. Given the salience of theoretical orientations on telehealth research questions and methodologies [16], we adopted the normalisation process theory (NPT), which would allow us to analyse the data systematically and coherently and present the findings. The NPT has been developed specifically for studying and describing the implementation of complex interventions within healthcare systems. It originated from a telehealth study in the United Kingdom to explore the processes by which new or modified practices become incorporated into routine practice [17]. This is different from other complementary theories used in telehealth research, such as the technology acceptance model (TAM) [18], that are adapted from other fields.

The NPT is a sociological theory that attempts to explain the factors influencing the implementation, embedding and integration of practices within the context of available resources [19]. It focuses on collective action in the implementation process. It has four key constructs. *Coherence* refers to individuals′ efforts to make sense of and invest meaning in a new practice. In *cognitive participation*, people commit themselves by enrolling in an implementation process and establishing its legitimacy. *Collective action* refers to people working individually and together towards implementation and is related to the workability and integration of the intervention into everyday practice. Through *reflexive monitoring*, individuals appraise the intervention′s effects [20]. The NPT has been successfully employed in the implementation research of complex healthcare interventions [21]. A systematic review in 2018 identified 130 published papers that utilised the NPT in studies of implementation processes across a wide range of healthcare and other interventions [21].

A mixed methods approach is appropriate in assessing the implementation of telepsychiatry, a complex intervention within a healthcare ecosystem that involves various factors that influence the process and outcome [22–24]. The availability of a validated NPT‐based questionnaire, the Normalisation Measures Development (NoMAD) instrument [25], makes the theory suitable for theory‐informed mixed methods evaluation studies of complex interventions, such as telepsychiatry in private practice.

In this study, we explored the normalisation of telepsychiatry in private practice in Australia. We aimed to (a) measure the degree of normalisation among private practice psychiatrists using the NoMAD instrument [25], (b) investigate clinician demographic and practice variables associated with routine use, (c) explore private practice psychiatrists′ experiences with telepsychiatry and (d) integrate the quantitative survey and qualitative interview findings.

## 2. Methods

The framework for Good Reporting of a Mixed Methods Study (GRAMMS) [26] and the American Psychological Association′s Mixed Methods Article Reporting Standards (MMARS) [27] guided this report.

### 2.1. Study Design

**Figure 1:**
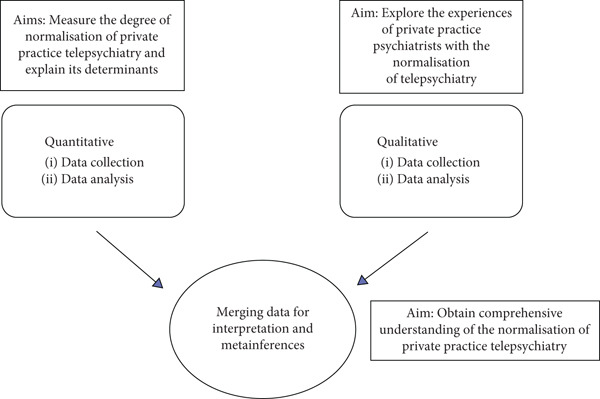
Convergent design of the mixed methods study of the normalisation of private practice telepsychiatry in Australia.

This convergent, parallel mixed methods study [22, 28, 29] consisted of a quantitative cross‐sectional survey allowing objective measures and generalisable findings and qualitative semistructured in‐depth interviews exploring personal experiences and perspectives [30] (Figure 1). Data integration occurred during the interpretation phase [22].

### 2.2. Researcher Description

The research team consisted of a psychiatrist and PhD candidate (L.S‐C.W.), two senior academic psychiatrists (J.C.L.L. and P.A.M.) and a senior mental health occupational therapist and researcher (R.E.R.). Together, we had substantial experience conducting quantitative, qualitative and mixed methods research.

### 2.3. Study Population

The study population was Australian private practice psychiatrists. The eligibility criteria were (a) registered with the Royal Australian and New Zealand College of Psychiatrists (RANZCP) and (b) current private practice in Australia (full‐time or part‐time). Australian psychiatrists practising overseas and psychiatric trainees were excluded.

### 2.4. Study Sample and Sampling

**Figure 2:**
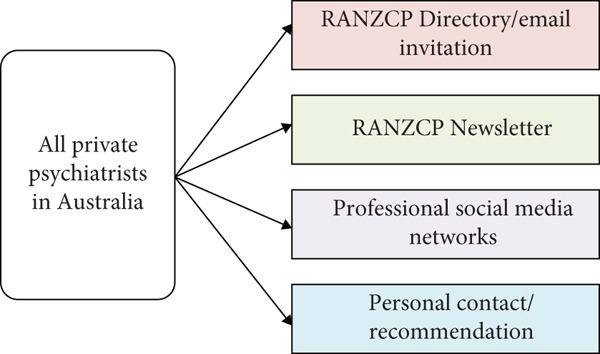
Sampling strategies of the study.

We surveyed using Qualtrics XM (Qualtrics, Provo, UT) from September 2023 to June 2024. Dissemination of the survey link to the study population occurred through several channels: email invitations to RANZCP members in its directory, private practice psychiatrists′ social media networks, the RANZCP Newsletter and face‐to‐face engagements at professional meetings. The sampling strategies are illustrated in Figure 2. Psychiatrists who met the eligibility criteria and consented to the study were included as study participants.

For the qualitative interviews, we took a targeted approach to include psychiatrists with different characteristics to obtain a wide range of input on the topic using nonrandomised purposive sampling [31]. We identified potentially suitable interviewees through the RANZCP directory of private psychiatrists, personal contacts and recommendations by other psychiatrists. We sought to recruit participants across different locations and diverse backgrounds (age, gender, subspecialty/area of interest, practice type and telepsychiatry usage level). Direct email invitations were sent to identify participants. Individuals who returned signed informed consent forms were included. In an iterative process, we coded and analysed the data while collecting data [32]. Participant recruitment continued until data saturation was achieved and no new materials emerged from the interview.

### 2.5. Study Instruments

We used the 23‐item NoMAD survey instrument (Supporting Information: NoMAD questionnaire) and collected demographic and clinical practice information (including telepsychiatry patient load). The NoMAD demonstrated adequate construct validity and internal consistency [25]. It assesses healthcare intervention implementation based on the NPT. It contains three general items and 20 items for four NPT subscales: coherence (four items), cognitive participation (four items), collective action (seven items) and reflexive monitoring (five items).

The general questions have a scale of 0–10 (with higher scores more favourable to normalisation), exploring if the intervention (1) feels familiar, (2) currently feels part of the normal work and (3) if it will become part of the normal work. The remaining questions have two response categories. Option A has five choices ranging from *strongly disagree* to *strongly agree*. One of the three choices in Option B can be selected instead if the question is irrelevant (to the respondent′s role, the intervention or the implementation stage). We used a 5‐point Likert scale for all Option A responses (1 = *strongly disagree*, 2 = *disagree*, 3 = *neither agree nor disagree*, 4 = *agree*, 5 = *strongly agree*) applied previously [33, 34], with Item 2 for collective action reverse‐coded.

We used an NPT‐based topic guide adapted from Scott et al. [35] for the semistructured interviews (Supporting Information: topic guide for semistructured interviews). L.S‐C.W. conducted the interviews using a secure videoconferencing platform. Interviews were audio‐recorded (with participants′ consent), transcribed verbatim and reviewed by interviewees.

### 2.6. Data Analysis

We performed quantitative analyses using Stata 18 (Statacorp LLC, College Station, TX). Descriptive statistics were generated. We examined Cronbach′s alpha values for internal consistency. Scores were calculated for NoMAD subscale items without missing Option A responses. No score was given to Option B responses. We calculated the average NPT subscale scores. The subscale summed scores were averaged over the items with valid Option A responses [34].

A dichotomous telepsychiatry clinical load variable was created by converting the multiple‐category responses to the ‘proportion of telepsychiatry practice’ question into ‘high’ patient load (proportion of > 50%) and ‘low’ (all other responses). Between the high and low groups, average NoMAD domain scores were compared using the Mann–Whitney *U* tests. Additionally, we recoded NoMAD responses as affirmative (*agree* or *strongly agree*) or nonaffirmative (*neither agree nor disagree*, *disagree*, *strongly disagree*, *not relevant* or *missing*). Affirmative responses in each subscale were summed and compared across telepsychiatry load groups. We also used chi‐square tests to compare each dichotomous NoMAD response between telepsychiatry load groups.

Subsequently, we performed a binary logistic regression analysis of demographic (age, gender and country of origin) and practice (years of experience, practice hours, practice type and practice area) characteristics of study respondents in high‐ and low‐telepsychiatry load groups for telepsychiatry consultations. The statistical significance level was set at 0.05.

We used NVivo 14 software (QSR International Pty Ltd., 2020) for thematic analysis [36] based on the a priori theoretical framework, NPT. The analysis was conducted by two members of the research team (L.S‐C.W. and R.E.R.) according to the six phases described by Braun and Clarke [36]. First, the coders read and reread the transcribed collected data to familiarise themselves with the dataset. In this process, we actively sought meanings and patterns in the data within the study′s context and theoretical framework. Initial codes were generated through the adaptation of a codebook from May et al. [37] (Supporting Information: codebook). We systematically worked through the dataset to collate data relevant to each code. During the coding process, we reviewed how the themes worked in relation to the coded extracts and the entire dataset. Iteratively, we refined the definitions of the themes to present a clear, coherent narrative. Lastly, vivid, illustrative coded extracts that were representative, transparent and fair were selected for inclusion in the report to provide a ‘thick description’ of the findings [38, 39].

We calculated the interrater reliability using Cohen′s kappa coefficients [40]. We discussed divergent views for kappa coefficients less than +0.81 to achieve reconciliation, clarifying codes′ meanings and adapting the codebook as needed [41]. The final kappa values ranged between +0.81 and +1.0, indicating ‘near‐perfect agreement’ [42].

Finally, we integrated the findings to assess the extent to which qualitative and quantitative results converge [43, 44] using a joint display [30, 45]. We drew metainferences [46] and described the added value from mixed methods that would not be available if only one form of data was used [47].

### 2.7. Ethical Considerations

This study received ethics approval from the Australian National University Human Research Ethics Committee (Protocol 2023/352). All study participants provided informed consent.

## 3. Results

### 3.1. Quantitative Findings

There were 110 survey respondents with valid responses (median age: 55, IQR: 45–63), with the median years of experience at 12 (IQR: 8–25). Table 1 shows the respondents′ demographic and practice characteristics.

**Table 1:** Characteristics of survey respondents (*n* = 110).

**Variable**	**n** **(%)**
Role	
Delivering telepsychiatry	105 (95.5%)
Managing or overseeing telepsychiatry	3 (2.7%)
Missing	2 (1.8%)
Gender	
Female	49 (44.5%)
Male	60 (54.5%)
Prefer not to say	1 (0.9%)
Country of origin	
Australia	65 (59.1%)
Overseas	43 (39.1%)
Missing	2 (1.8%)
Work hours	
Full‐time	36 (32.7%)
Part‐time	73 (66.4%)
Missing	1 (0.9%)
Practice type	
Solo practice	74 (67.3%)
Group practice	28 (25.5%)
Others	8 (7.3%)
Practice area	
Metropolitan	70 (63.6%)
Nonmetropolitan	15 (13.6%)
Both	24 (21.8%)
Missing	1 (0.9%)
State/territory^a^	
ACT	5 (4.5%)
NSW	44 (40.0%)
QLD	31 (28.2%)
SA	5 (4.5%)
TAS	2 (1.8%)
VIC	30 (27.3%)
WA	9 (8.2%)
Duration of telepsychiatry practice	
< 1 year	6 (5.5%)
≥ 1–< 3 years	32 (29.1%)
≥ 3–< 10 years	57 (51.8%)
≥ 10 years	13 (11.8%)
Missing	2 (1.8%)
Frequency of telepsychiatry practice	
Daily	44 (40.0%)
At least once a week	56 (50.9%)
At least once a month	9 (8.2%)
Less than once in 3 months	1 (0.9%)
Proportion of telepsychiatry practice	
< 5%	8 (7.3%)
5%–< 10%	12 (10.9%)
10%–< 20%	21 (19.1%)
20%–< 50%	27 (24.5%)
≥ 50%	42 (38.2%)

Abbreviations: ACT, Australian Capital Territory; NSW, New South Wales; QLD, Queensland; SA, South Australia; TAS, Tasmania; VIC, Victoria, WA, Western Australia.

^a^More than one response allowed.

#### 3.1.1. NoMAD Responses

**Figure 3:**
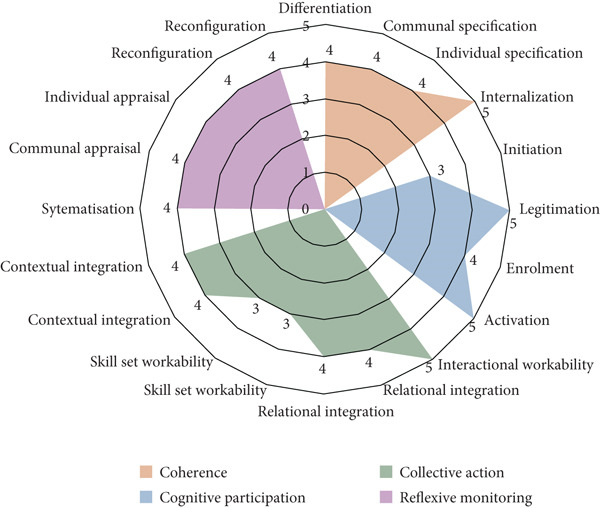
Petal chart depicting the median scores for responses to NoMAD items. Each of the following are represented by two NoMAD items: relational integration, skill set workability, contextual integration and reconfiguration.

Table 2 and Figure 3 provide information for the NoMAD responses. The overall internal consistency of all specific items (excluding general items) was high (Cronbach^′^s alpha = 0.90). The subscales also displayed high internal consistency except for coherence (Cronbach^′^s alpha = 0.46). Table S1 shows the breakdowns of Option A responses to NoMAD items.

**Table 2:** Summary of responses to NoMAD items.

**NoMAD component**	**No. of items**	**Cronbach′s alpha (standardised)**	**Average score, median (IQR)**	**Summed positive response, median (IQR)**
How familiar is telepsychiatry, *n* = 105	1	—	9.0 (8.0–10.0)	—
Is telepsychiatry currently a normal part of work, *n* = 107	1	—	10.0 (8.0–10.0)	—
Will telepsychiatry become a normal part of work, *n* = 103	1	—	10.0 (8.0–10.0)	—
Coherence items	4	0.455	4.2 (4.0–4.7)	3.0 (3.0–4.0)
Cognitive participation items	4	0.715	4.3 (4.0–4.8)	3.0 (2.0–4.0)
Collective action items	7	0.837	3.7 (3.3–4.2)	3.0 (2.0–5.0)
Reflexive monitoring items	5	0.807	4.0 (3.5–4.8)	4.0 (3.0–5.0)

The general items, average cognitive participation score and average reflexive monitoring score had statistically significant greater values for the high telepsychiatry load group (Table 3). The statistical test results for summed positive responses of cognitive participation and reflexive monitoring resembled the average scores. Coherence was statistically insignificant, but collective action was statistically significant.

**Table 3:** Comparisons of NoMAD response between high‐ and low‐telepsychiatry load groups.

	**Clinical load**		**p** **value**
	**Low, median (IQR)**	**High, median (IQR)**	
Average score			
How familiar is telepsychiatry	9.0 (7.0–10.0)	10.0 (9.0–10.0)	< 0.001 ^∗^
Is telepsychiatry currently a normal part of work	9.0 (7.0–10.0)	10.0 (10.0–10.0)	< 0.001 ^∗^
Will telepsychiatry become a normal part of work	9.0 (7.0–10.0)	10.0 (10.0–10.0)	< 0.001 ^∗^
Coherence	4.1 (4.0–4.3)	4.4 (4.0–4.8)	0.258
Cognitive participation	4.0 (3.8–4.7)	4.5 (4.3–4.8)	< 0.034 ^∗^
Collective action	3.5 (3.1–3.8)	4.1 (3.6–4.5)	0.177
Reflexive monitoring	4.0 (3.4–4.6)	4.5 (3.6–5.0)	< 0.001 ^∗^
Summed affirmative responses			
Coherence	3.0 (3.0–4.0)	3.0 (3.0–4.0)	0.894
Cognitive participation	3.0 (2.0–3.0)	3.0 (3.0–4.0)	< 0.001 ^∗^
Collective action	3.0 (1.5–4.0)	5.0 (3.0–7.0)	< 0.001 ^∗^
Reflexive monitoring	3.5 (2.5–5.0)	4.0 (3.0–5.0)	0.042 ^∗^

^∗^Statistically significant.

When NoMAD dichotomous responses (affirmative and nonaffirmative) were compared, the high‐load group was *less likely* to view telepsychiatry as different from usual work practice in the coherence subscale. This group was more likely to endorse all cognitive participation items. It was similar for most collective action items, except for the reverse‐coded item (‘telepsychiatry disrupts working relationships’). Both groups similarly highly endorsed the item on the ease of integrating telepsychiatry into existing work. For reflexive monitoring, the high‐load group was more likely to be aware of the reports on telepsychiatry′s effects and appreciative of telepsychiatry′s value (Table S2).

#### 3.1.2. Demographic and Practice Variables

Binary logistic regression analysis revealed that solo practices were six times more likely to have a high telepsychiatry load than group practices (OR: 6.347, 95% CI: 1.492–27.026). Private practices covering nonmetropolitan areas (OR: 7.045, 95% CI: 1.415–35.086) and both metropolitan and nonmetropolitan areas (OR: 8.273, 95% CI: 2.1093–32.450) were substantially more likely than metropolitan‐only private practices to have a high telepsychiatry load. Although *years of experience* as an independent variable yielded a statistically significant result, there was little ‘real word significance’ given the odds ratio of 1.125 (Table 4).

**Table 4:** Results of the logistic regression analysis of demographic and practice characteristics for telepsychiatry clinical load.

**Variable**	**OR**	**SE**	**p** **value**	**95% CI**
Age	0.919	0.047	0.102	0.831, 1.017
Gender^a^				
Female (referent)				
Male	0.419	0.249	0.143	0.131, 1.342
Country of origin^b^				
Australia (referent)				
Overseas	1.040	0.562	0.943	0.361, 3.000
Years of experience	1.125	0.058	0.022 ^∗^	1.017, 1.244
Private practice hours^c^				
Full‐time (referent)				
Part‐time	1.300	0.759	0.653	0.414, 4.081
Private practice type				
Group practice (referent)				
Solo practice	6.347	4.692	0.012 ^∗^	1.491, 27.026
Others	7.721	8.633	0.068	0.863, 69.086
Practice area				
Metropolitan (referent)				
Nonmetropolitan	7.045	5.771	0.017 ^∗^	1.415, 35.086
Both	8.273	5.769	0.002 ^∗^	2.109, 32.450
Constant	7.316	15.359	0.343	0.119, 448.068

^a^The category ‘prefer not to say’ (one observation) was omitted from the model.

^b^The category ‘missing’ (two observations) was omitted from the model.

^c^The category ‘missing’ (one observation) was omitted from the model.

^∗^Statistically significant.

### 3.2. Qualitative Findings

**Figure 4:**
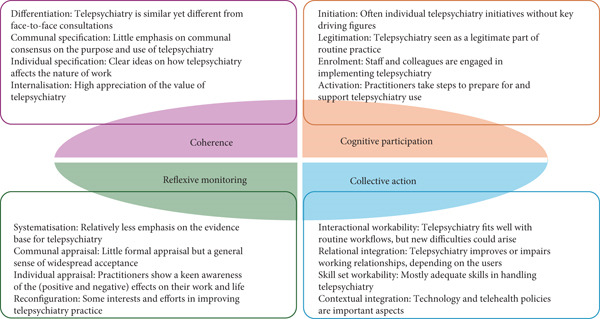
Main findings from the qualitative analysis according to the NPT constructs.

Thirteen participants from diverse personal and professional backgrounds were interviewed between January and May 2024 (Table S3). Figure 4 summarises the main findings.

#### 3.2.1. Coherence

*Differentiation*: For most practitioners, the differences between telepsychiatry and face‐to‐face consultations are negligible and do not hinder an effective consultation.

When the system works well, very little difference from my point of view. So, if you have a good quality video feed, I′m able to connect with someone. (P1)

I don′t think it′s different, to be honest… I have been a face‐to‐face psychiatrist for most of my career, and now I just do telehealth. I don′t see any difference other than, you know, I think it′s a safer way to practice psychiatry. (P10)

However, for some, telepsychiatry might result in reduced information from mental state examination, particularly for telepsychiatry via telephone.

And in a lot of cases anyway, I find I′m better able to form a deeper connection, a deeper therapeutic alliance when I′m sitting in a room with a person. It just feels a little bit less removed. So, that′s why I prefer face‐to‐face reviews. (P8)

*Communal specification*: In some group practices, practitioners discuss and achieve consensus on the purpose and use of telepsychiatry. However, in many cases, a communal understanding of telepsychiatry is not a priority, especially among solo practitioners.

We never actually discussed [telepsychiatry]. Because we were separate practices. It wasn′t like a group practice where we covered each other′s patients or anything… It just never came up. (P2)

*Individual specification*: Practitioners recognise and envision that telepsychiatry helps improve the accessibility of psychiatric consultations in their private practices.

It makes it easier for people to access sessions when they are in situations where it′s harder to be mobile or go to a physical clinic. The population of patients that I mainly see is women who want to be pregnant, who are pregnant, or who are quite soon postpartum… for women with young babies, or in late pregnancy, it is usually easier to not have to travel and leave the house. (P3)

Some practitioners emphasise the need to offer both telepsychiatry and face‐to‐face consultations flexibly.

We would let the patient know right from the outset of referral or intake, that they have the option to come in person or via telehealth, preferably initial assessment will be in person. Then, they would be told about the ways to get access to telehealth. (P9)

On the downside, expanded coverage via telepsychiatry also presents an increased likelihood of unfamiliarity with the patients′ local healthcare settings.

It meant that my referrals tended to come from interstate sometimes or further away… which wouldn′t normally happen otherwise. And therefore, I was a bit careful that when I screened the referrals, if there was anything that I thought was too acute or too dangerous, and couldn′t wait… I wouldn′t be able to help much, because I′m only doing telehealth…. (P3)

*Internalisation*: Practitioners perceive telepsychiatry as valuable in various aspects of clinical practice and career, including patient attendance, safety and flexibility.

I think the presence of telehealth has increased attendance rates dramatically at consultations. (P5)

I think it′s a safer way to practice psychiatry. I think the risk of things like boundary violations is completely or pretty much removed. (P10)

From the practitioner′s point of view, it might encourage me to continue to practice a little longer than I might otherwise have planned. (P12)

#### 3.2.2. Cognitive Participation

*Initiation*: Sometimes, a psychiatrist with previous experience and/or special interest in telepsychiatry gets other colleagues involved in its usage.

He′s the principal psychiatrist of that practice. He′s been instrumental in being the architect of that service… So, he′s been the main driver of [telepsychiatry]. (P8)

It is most often up to individual practitioners to decide if they wish to use telepsychiatry.

No. There′s nobody who′s… when you run into them in the tearoom saying, ‘Gosh! This telepsychiatry is amazing. No. (P2)

*Legitimation*: Improved patient access was the chief argument for the legitimacy of maintaining telepsychiatry services in private practice.

It gives patients a lot more autonomy, and for many of them who might be fearful of things… or have difficulty travelling, it opens things up that were not available before. (P13)

Still, there is a concern that the misuse of telepsychiatry might jeopardise its place in routine clinical practice.

The main concern… is the risk of ‘cowboys’ doing telehealth, bringing it into disrepute, and the government withdrawing funding… It′s the risk that the ‘cowboys’… damage the whole thing for everybody, particularly the patients. (P10)

*Enrolment*: Practitioners observe that most staff members are willing and able to support telepsychiatry effectively after their initial exposure.

My administrative staff are amazing and keep me on track… Part of their responsibilities is making sure the patient has got today′s link. If they′re not there on time, they might contact and see if they′re having problems with the technical aspect…. (P1)

Others noticed that less tech‐savvy supporting staff can find it quite difficult to adjust to telepsychiatry.

Sometimes we have one of our receptionists who′s a bit older, so she′s not necessarily the most tech‐savvy person, and not necessarily the best at helping patients troubleshoot if something doesn′t go well. (P4)

*Activation*: Practitioners′ efforts in planning and preparation often focus on telepsychiatry platforms and hardware and compliance with regulatory requirements, including medical indemnity.

I did go on to the RANZCP website to try to look at if they had any guidelines about privacy and that kind of stuff with telehealth. And I think I also looked up AHPRA (Australian Health Practitioner Regulation Agency)… and my practice indemnity insurance…. (P3)

This phase can be challenging for practitioners with little prior experience with telepsychiatry and insufficient support.

#### 3.2.3. Collective Action

*Interactional workability*: The adoption of telepsychiatry works well with components of consultations in private practice and often enhances efficiency in areas like assessments and psychoeducation.

I can assess someone′s affect, and convey empathy using an audiovisual technology just as well as in person… It′s probably more comprehensive because the summaries of the report measures are displayed in a panel on the screen… I can be talking to the patient, and I can access the record at the same time…. (P6)

A drawback is that handling unexpected situations while interacting with patients using telepsychiatry can be more difficult.

And what [the practitioner] didn′t realise was that throughout all the later sessions, the patient′s partner was standing in the room just out of sight of the camera. So, you don′t have control of the environment…. (P2)

*Relational integration*: Telepsychiatry creates more ways to engage patients, caregivers and other healthcare professionals. There is confidence in others′ ability to use telepsychiatry.

You can engage a lot of people all at once. So, the behavioural support worker or the analyst, the guardian, the parents, the NDIS (National Disability Insurance Scheme) coordinator, the routine support worker, and the local GP can dial in all at once in that one hour and come to a consistent consensus without making so many calls and so many correspondences to solve an issue… You get a little window into [a patient′s] life… And you get a little bit more idea about who they are, especially if they′re at home. They seem to have more openness about them. (P9)

Nevertheless, a predominant or exclusive telepsychiatry practice may result in an increased sense of professional isolation and diminished collegiality among the practitioners.

I think there can be an element of being siloed when it′s just you and your desk… That regular face‐to‐face with colleagues means these little ideas or little hallway discussions, ‘Oh, I saw this patient, and I want your opinion…’ I think there′s a little bit less collegiality unless you really go seeking it… because there is the temptation to just work from home. (P8)

Additionally, telepsychiatry may not work well with some people (old age, sensory impairment, language barrier, etc.).

Now, some people aren′t suitable… Obvious people like, where you do not speak their same language…. People who have got hearing impairment and require a signing interpreter… They′ve probably pretty much got to be done face‐to‐face. (P7)

*Skill set workability*: Practitioners often already possess general videoconferencing skills. They can demonstrate their ability to conduct telepsychiatry consultations thoughtfully and safely.

I ask my patients to get set up with privacy as well. I have a little checklist… So, in the unlikely event of an emergency, if I need to call emergency services, I need an address to be able to send them to… I′ll ask them what′s the setting. And I′ll ask them for an emergency contact number. So, if it′s a teenager, it′ll usually be a parent. If it′s a young adult, it might be a parent or a partner…. (P11)

Sometimes practitioners express inadequate self‐confidence in doing telepsychiatry, especially at the initial stage. The need to develop telepsychiatry‐specific skills is also pointed out.

We were never trained in telehealth… This is something that emerged out of COVID‐19. So, thinking about how to train emerging clinicians, not only in traditional clinical skills face‐to‐face but developing some skills in doing it online should be part of a curriculum going forward because it′s the future. (P6)

*Contextual integration*: Advances in technology and innovations in administrative processes have made telepsychiatry considerably easier to implement. Electronic prescription, or eScript, was an example cited.

The most difficult issue was prescribing, so until eScripts came along, it was quite difficult to prescribe… It was an enormous amount of work and cost to do all that. That has gone away with eScripts has just revolutionised that. Now it′s extremely easy to prescribe. (P10)

However, problems with the technology can still be a recurring issue. On the financial aspect, expanded reimbursements for telepsychiatry help greatly, but changing funding policies can add uncertainty and barriers.

#### 3.2.4. Reflexive Monitoring

*Systematisation*: A few practitioners are cognisant of and keep themselves up‐to‐date with the clinical evidence for telepsychiatry in published research. However, many practitioners do not actively and systematically appraise the scientific evidence for telepsychiatry.

I must admit I haven′t really looked into the academic research about it. I′ve just used it… as a practical tool. I have just always assumed, from my own experience, that it is probably slightly inferior to seeing someone in person, but that that′s balanced out by increasing people′s access and convenience. (P4)

*Communal appraisal*: While formal appraisals of telepsychiatry in a communal setting among practitioners are rare, there is a sense that telepsychiatry is widely accepted by all parties involved, including doctors.

I think all doctors, all patients, all receptionists, are very familiar with telehealth and how to explain it to patients… I think there′s widespread acceptability. (P5)

*Individual appraisal*: Many practitioners see the clinical benefits experienced by their patients who access telepsychiatry. They also appreciate the positive effects of telepsychiatry on their practice and personal life, notably the flexibility that it affords, which can make working with patients easier (e.g., improved clinic attendance) and better quality of life.

The vast majority of my patients say that [telepsychiatry] is so much better for them. (P13)

It provides the sort of continuity in terms of prioritising care and making sure that patient gets the care, and you′re rewarded financially for dedicating the time for that patient…. (P9)

I′m able to juggle my work‐life balance very effectively, which means I′m more likely to do a Saturday clinic because I know that I get some downtime afterwards without having to get in the car and travel… an opportunity to enjoy the work‐life balance and grow the business. (P6)

Meanwhile, some also observe that telepsychiatry practice can have its downsides, for instance, a blurring of boundaries between work and personal life.

[Telepsychiatry affects my work‐life balance] probably not in a good way, because it′s just so much easier to book another day′s clinic, or to follow up with out‐of‐hour emails and stuff like that than I otherwise would. (P1)

*Reconfiguration*: Improvements in telepsychiatry have been considered and attempted in clinical and technical aspects, yet many have not thought of modifying their telepsychiatry practices specifically.

I′m constantly trying to improve it and make it better… I′ve developed a template email for the medications that I might prescribe on a relatively regular basis… I can click a button, and the patient gets emails, some information which is a combination of the consumer medication information, and my advice on the medication they′re about to start taking. (P10)

### 3.3. Integration

In the joint display (Table 5), we included two aspects of the quantitative survey: the average scores for the NPT subconstructs (subthemes) and differences in responses in the high telepsychiatry load group. We also incorporated the qualitative subthemes and their relative emphasis (as indicated by the subtheme coding frequency).

**Table 5:** Joint display for the integration of quantitative and qualitative findings.

**Theme**	**Quantitative**		**Qualitative**		**Analytical integration**
	**Average score**	**High-load group,** **p** **value** ^**a**^	**Summary**	**Emphasis, reference counts** ^**b**^	
Coherence					
Differentiation	4	Less positive, 0.003	Telepsychiatry is largely seen as equivalent to face‐to‐face consultations, but it can be different from face‐to‐face consultations, especially in the extent the clinician can observe the patient	Moderate, 66	Partial convergence across quantitative and qualitative findings In the survey, respondents often saw telepsychiatry as different However, respondents with high telepsychiatry load tended to agree with the dominant view in interviews that it is similar to face‐to‐face consultations
Communal specification	4	0.081	Communal specification is not relevant in solo practices. In group practices/practices with more than one practitioner, conscious efforts to specify the use of telepsychiatry are often absent. Dedicated telepsychiatry practices could be exceptions	Low, 39	No convergence across quantitative and qualitative findings Survey respondents reported a high level of shared understanding of telepsychiatry′s purpose among staff members Interviews revealed that this aspect was often not explicitly discussed, as many were in solo practices
Individual specification	4	0.475	Practitioners often have clear ideas about the ways telepsychiatry affects the nature of their work, mostly in positive ways	High, 98	Convergence across quantitative and qualitative findings In the survey, respondents recognised the effects of telepsychiatry on their work, regardless of telepsychiatry patient load Qualitative interviews indicated that the perceived effects were largely positive
Internalisation	5	0.121	Practitioners highly appreciate the value of telepsychiatry in their work, in the way they manage and run their practices	High, 77	Convergence across quantitative and qualitative findings In the survey, respondents affirmed the value of telepsychiatry in their work, regardless of telepsychiatry patient load Qualitative interviews revealed the perceived value of telepsychiatry on practitioners′ careers and for their patients
Cognitive participation					
Initiation	3	More positive, 0.007	Rarely there is a key person who drives the use of telepsychiatry at private practices, but in some group practices, a senior clinician′s advocation for telepsychiatry is influential	Low, 19	Partial convergence across quantitative and qualitative findings In both the survey and interviews, the role of a key person driving telepsychiatry′s usage was not prominent However, having a key person was likely to be associated with higher telepsychiatry loads in the practices
Legitimation	5	More positive, 0.004	Most practitioners see telepsychiatry as having a legitimate place in private practice given its substantial benefits to patients	Moderate, 55	Convergence across quantitative and qualitative findings In both the survey and interviews, the legitimacy of telepsychiatry was recognised and was linked to higher usage The interviews revealed both positive (improved patient access) and negative issues (misuse) related to telepsychiatry
Enrolment	4	More positive, 0.001	When needed, practitioners can engage and collaborate with supporting staff to begin new telepsychiatry services	Low, 26	Partial convergence across quantitative and qualitative findings The survey showed that respondents were open to collaborating with others in using telepsychiatry Despite lesser emphasis, the interviews revealed that the practitioners could engage staff members in implementing telepsychiatry
Activation	5	More positive, 0.032	Practitioners show various levels of planning and preparation to support telepsychiatry in their practices	Moderate, 31	Convergence across quantitative and qualitative findings In the interviews, high levels of support for telepsychiatry were associated with greater use The interviews highlighted aspects of planning and preparation that reflected practitioners′ support for telepsychiatry
Collective action					
Interactional workability	5	0.081	Telepsychiatry can be readily integrated into routine workflow in private practices but also presents new challenges	Moderate, 49	Convergence across quantitative and qualitative findings Survey respondents highly affirmed telepsychiatry′s interactional workability The interviews revealed the ways telepsychiatry can fit into and enhance existing workflows
Relational integration^c^	4	More positive, < 0.001, 0.040	Telepsychiatry improves the practitioners′ capability to work with other healthcare professionals, but not all parties involved are similarly competent. It can also lead to professional isolation sometimes	High, 76	Convergence across quantitative and qualitative findings In the survey, working well with others by telepsychiatry was linked to more usage The interviews revealed the ways telepsychiatry can enhance collaboration with others in patient care
Skill set workability^c^	3	More positive, 0.004, < 0.001	Practitioners often acquire the skills (IT, interviewing, etc.) required for telepsychiatry consultations relatively easily	Moderate, 59	Partial convergence across quantitative and qualitative findings In the survey, telepsychiatry‐related skills/training were linked to more usage The interviews highlighted skills and confidence in using telepsychiatry
Contextual integration^c^	4	More positive, < 0.001, 0.030	Appropriate equipment and software, technical support and administrative changes (prescribing, billing, etc.) are among the contextual factors that affect the implementation of telepsychiatry	High, 80	Convergence across quantitative and qualitative findings In the survey, telepsychiatry‐related resources and support were linked to more usage The interviews revealed the types of resources and administrative support needed for telepsychiatry
Reflexive monitoring					
Systematisation	4	More positive, 0.045	Practitioners generally do not emphasise the evidence base for telepsychiatry′s efficacy from the literature but rather see it just as a convenient tool and alternative to face‐to‐face consultations	Low, 22	No convergence across quantitative and qualitative findings The survey result indicated a general understanding of telepsychiatry′s effects, with greater awareness linked to higher usage In the interviews, practitioners seldom regarded telepsychiatry as a distinct intervention requiring its own evidence base
Communal appraisal	4	0.357	Agreement with peers concerning the effects and outcomes of telepsychiatry is infrequently mentioned	Low, 23	No convergence across quantitative and qualitative findings The survey result indicated a general impression about a positive consensus on telepsychiatry In the interviews, practitioners infrequently reported efforts for collective appraisals of telepsychiatry′s effects in their practices
Individual appraisal	4	More positive, 0.012	Practitioners personally extensively review and appreciate the (generally positive) impacts of telepsychiatry	High, 96	Convergence across quantitative and qualitative findings In the survey, telepsychiatry was valued, especially by frequent users The interviews revealed the aspects of telepsychiatry valued by the practitioners
Reconfiguration^c^	4	0.176, 0.768	Some practitioners propose ways to improve their telepsychiatry services based on their experiences and feedback	Low, 21	No convergence across quantitative and qualitative findings The survey result indicated a readiness to adjust how telepsychiatry is used in practice In the interviews, modifications of current telepsychiatry practice less emphasised but some have proposed meaningful changes

^a^For the Chi‐square test, comparing the dichotomous responses to NoMAD items with the low‐telepsychiatry load group.

^b^Count of references to the subtheme in the interview transcripts as coded by two coders (low ≤ 30, moderate 30–70 and high > 70).

^c^Consisted of two NoMAD items.

We drew several inferences:

• In making sense of telepsychiatry as a new practice, perceived similarity to conventional face‐to‐face consultations was a positive factor. There was an appreciation of telepsychiatry′s potential in private practice.
• In the private practice environment, adopting telepsychiatry is more likely to be a ‘bottom–up’ decision rather than a ‘top–down’ process. Greater personal commitments encourage telepsychiatry implementation. In group practices, senior practitioners can influence telepsychiatry′s uptake.
• While most practitioners recognise that telepsychiatry can work well, confidence in oneself, others and the support systems for telepsychiatry usage are especially important in its adoption and routine use.
• Practitioners have a general impression and understanding of telepsychiatry′s effects in their practices, even though in‐depth appraisals might be absent. A greater individual appreciation of its positive impacts may be valuable in maintaining usage.

## 4. Discussion

In this study, we explored telepsychiatry′s normalisation among Australian private practice psychiatrists amidst pandemic‐driven increased availability and demand. Our survey found a high level of familiarity with telepsychiatry. Higher cognitive participation and collective action were associated with greater use. The high‐load group was less likely to view telepsychiatry as different from usual work practice and more likely to be aware of telepsychiatry′s effects. Additionally, being a solo practice and a practice covering nonmetropolitan areas were associated with higher telepsychiatry loads. We mapped the qualitative findings to the NPT constructs. Lastly, we integrated the quantitative and qualitative results and drew several inferences.

Several positive and negative aspects of telepsychiatry were mentioned. Telepsychiatry was largely regarded as equivalent to conventional face‐to‐face consultations, similar to findings in recent meta‐analyses [48, 49]. It could facilitate communication, improve accessibility and flexibility and enhance the efficiency of existing workflows, benefiting clinicians and patients [50]. Supportive policy frameworks, resource availability and widespread acceptance ease telepsychiatry adoption. Conversely, there were concerns that nuanced aspects of the doctor–patient interaction and assessments, such as maintaining a good flow of communication during consultation and establishing rapport, could be impaired [10, 13], although it worked well on most occasions [5, 6]. Telepsychiatry practice might, however, lead to professional isolation and blurring of work–life boundaries. Inadequate support, resources and skills might exclude some psychiatrists and patients from telepsychiatry. Telepsychiatry may not be the best clinical practice for certain situations, such as emergencies, or patients with communication difficulties [8].

A key finding is that regular telepsychiatry users largely did not view telepsychiatry as a distinct form of intervention in their work. This is different from the original NPT differentiation concept, which emphasises the ability of the practitioners to ‘appreciate how it differs or is clearly distinct from current ways of working’ [51]. Regular users were more likely to affirm telepsychiatry′s potential and values and commit themselves to its implementation than less frequent users. In general, there were high levels of familiarity, acceptance and appreciation of telepsychiatry, similar to previous Australian studies [14], even among less frequent users in the survey. This was reflected by the uniformly high affirmation of coherence and reflective monitoring items/subthemes. Furthermore, telepsychiatry was seen as highly compatible with existing practice, contrary to an early NPT study more than two decades ago, which found telepsychiatry disruptive [52]. However, this accepting attitude did not necessarily translate into a strong initiative to use telepsychiatry. How much telepsychiatry is routinely used seems to be less due to perceptions and more related to the practical needs and characteristics of psychiatrists and their practices [13], for instance, to improve clinic attendance through offering telepsychiatry [9].

Private practices serving nonmetropolitan areas were much more likely than those covering major cities alone to have a high telepsychiatry patient load. This could be due to the accessibility‐related convenience of telepsychiatry for rural regions perceived by practitioners serving them. This practice factor for higher telepsychiatry usage concurs with practitioners′ notion that enhanced access was an important benefit of telepsychiatry (‘internalisation’) and a strong argument for its legitimacy. Previous studies also indicated the usefulness of telehealth for rural healthcare [53, 54], although there are also challenges with internet and equipment availability [8]. In this respect, research on telepsychiatry′s impact on rural health equity, including formulating policies to encourage telepsychiatry uptake in rural and remote areas, may provide further evidence for its usefulness.

Solo practices were more likely to have high telepsychiatry clinical loads. The relatively low telepsychiatry usage in group practices may be due to the lower emphasis on communal specification in the qualitative interviews. However, in the survey, the high‐load group had significantly greater communal specification than the low‐load group. Those with high telepsychiatry patient loads were more likely to affirm efforts to lead and engage staff for telepsychiatry. There may be two major factors in uptake. First, solo practitioners can initiate and implement telepsychiatry with relative ease. By lowering the threshold for telepsychiatry‐related skills, software and equipment, and increasing the availability of resources, including funding and billing support through Medicare, as well as streamlined online prescribing via eScripts, the maturing telehealth ecosystem allows an interested practitioner to set up and maintain telepsychiatry services independently. Our findings highlight the importance of legal, regulatory and reimbursement support in sustaining telepsychiatry use [4, 55]. Second, while the decision‐making process for telepsychiatry in group practices can be more complicated, having strong leadership advocating for telepsychiatry with clear communication and goal setting can provide the crucial push for its implementation [56].

Lack of specific skills may be an impeding factor for routine telepsychiatry usage. Infrequent users in the survey scored low for skill set workability. They were less likely to agree that there were adequate skills to implement telepsychiatry in their private practices. In the interviews, practitioners less confident in using telepsychiatry perceived themselves as possessing insufficient skills. Poor skills may cause low telepsychiatry usage and/or inadequate practice results in underdeveloped skills. While the general information technology skills needed to run videoconferencing sessions have become less demanding and easier to acquire, the acquisition of telepsychiatry‐specific clinical skills remains a relatively neglected area despite the benefits of telepsychiatry training [11, 15, 57]. The optimistic notion that telepsychiatry is no different from face‐to‐face consultations may gloss over subtly different approaches needed in telepsychiatry. For instance, some observations and physical examinations practicable in face‐to‐face consultations are unavailable in telepsychiatry, and the clinician must adjust the assessment accordingly. Meanwhile, some clinicians′ impression that telepsychiatry is certainly inferior may be at least partially due to unfamiliarity with the required skills. The availability of telepsychiatry‐related continuous professional development resources and training programmes is essential [13]. Moreover, telehealth clinical skills should become part of the core curriculum for medical students and psychiatry trainees [58, 59].

While acknowledging the large and growing literature in this area, to our knowledge, our mixed methods study was the first to investigate private practice telepsychiatry in Australia based on the NPT framework, producing useful insights through the combination of quantitative and qualitative findings. However, this study had several limitations. Although the survey was open to private psychiatrists nationally, the sampling was not random, and the sample size was relatively small. However, compared with general populations, surveys of special populations like this are less susceptible to nonresponse bias [60]. The percentage of solo practitioners in our study (66.4%) was moderately higher than the private psychiatrist data (53.5%; solo practitioners: 892, group practitioners: 776) reported by the Australian Institute of Health and Welfare [61]. The NoMAD instrument lacked a scoring system that generated an overall normalisation measure. Additionally, the telepsychiatry patient load was based on respondents′ self‐report. Finally, the researcher′s bias in interpreting the results of the qualitative analysis cannot be entirely excluded. While the national focus of the current study might limit the international generalisability of the findings, they provide a necessary foundation for subsequent comparative studies across countries.

In conclusion, telepsychiatry was perceived as a useful tool that can be successfully integrated into routine private practice with sufficient support. There was high acceptance and readiness for telepsychiatry implementation. While telepsychiatry may not be suitable for all practitioners and patients, it has a place in routine practice for many clinicians who recognise its benefits and potential within their contexts. Future research should examine initiatives to improve the acquisition of telepsychiatry‐related clinical skills, strategies to maintain or improve collegiality and service outcomes to inform legislative and administrative frameworks to support sustained integration of telepsychiatry in this sector.

## Ethics Statement

This study was conducted per the Declaration of Helsinki, with ethics approval from the Australian National University Human Research Ethics Committee (Protocol 2023/352). All study participants provided informed consent.

## Conflicts of Interest

The authors declare no conflicts of interest.

## Author Contributions

Luke Sy‐Cherng Woon: conceptualization, methodology, software, data curation, formal analysis and writing—original draft preparation. Paul A. Maguire: conceptualization, methodology, formal analysis, writing—reviewing and editing and supervision. Jeffrey C. L. Looi: conceptualization, methodology, writing—reviewing and editing and supervision. Rebecca E. Reay: conceptualization, methodology, formal analysis, writing—reviewing and editing and supervision.

## Funding

No funding was received for this manuscript.

## Supporting information


**Supporting Information** Additional supporting information can be found online in the Supporting Information section. NoMAD questionnaire. The following questionnaire was adapted from the original NoMAD instrument [62]. The corresponding author granted permission for the adaptation and use of the instrument. The changes were to ‘Part A: About Yourself’, which was tailored to respondents in psychiatry private practice in Australia, and the substitution of the generic term ‘the intervention’ with ‘telepsychiatry’ throughout the questionnaire. Supporting information: Topic guide for semistructured interviews. This is the topic guide used during the online semistructured interviews with private practice psychiatrists. Supporting information: Codebook: this is the codebook used for the coding process of the thematic analysis of semistructured interview content. Table S3: This table shows the characteristics of the participants in semistructured interviews. Table S2: This table shows the comparisons of dichotomous NoMAD responses between respondents with high and low telepsychiatry clinical load. Table S1: This table shows the Option A responses to NoMAD items.

## Data Availability

The data that support the findings of this study are available on request from the corresponding author. The data are not publicly available due to privacy or ethical restrictions.
