# Supplementary material for: Exploring the Normalisation of Telepsychiatry Practice Among Private Psychiatrists in Australia: A Convergent Mixed Methods Study
Source: Int J Telemed Appl. 2025 Dec 18;2025:4100418. doi: 10.1155/ijta/4100418 (PMC12752830; doi:10.1155/ijta/4100418)
Supplement: Supplementary file 1 — Supporting Information Additional supporting information can be found online in the Supporting Information section. NoMAD questionnaire. The following questionnaire was adapted from the original NoMAD instrument [62]. The corresponding author granted permission for the adaptation and use of the instrument. The changes were to ‘Part A: About Yourself’, which was tailored to respondents in psychiatry private practice in Australia, and the substitution of the generic term ‘the intervention’ with ‘telepsychiatry’ throughout the questionnaire. Supporting information: Topic guide for semistructured interviews. This is the topic guide used during the online semistructured interviews with private practice psychiatrists. Supporting information: Codebook: this is the codebook used for the coding process of the thematic analysis of semistructured interview content. Table S3: This table shows the characteristics of the participants in semistructured interviews. Table S2: This table shows the comparisons of dichotomous NoMAD responses between respondents with high and low telepsychiatry clinical load. Table S1: This table shows the Option A responses to NoMAD items. [file IJTA-2025-4100418-s001.docx]

# Supplementary materials

**Supplementary Table 1**

Supplementary Table 1 Option A responses to NoMAD items.

| Item | n (%) |
| --- | --- |
| I can see how telepsychiatry differs from usual ways of working |  |
| Strongly disagree | 3 (2.8%) |
| Disagree | 8 (7.4%) |
| Neither agree nor disagree | 10 (9.3%) |
| Agree | 59 (54.6%) |
| Strongly agree | 28 (25.9%) |
| Staff in this organisation have a shared understanding of the purpose of telepsychiatry |  |
| Disagree | 5 (6.2%) |
| Neither agree nor disagree | 6 (7.4%) |
| Agree | 36 (44.4%) |
| Strongly agree | 34 (42.0%) |
| I understand how telepsychiatry affects the nature of my own work |  |
| Neither agree nor disagree | 8 (7.3%) |
| Agree | 59 (53.6%) |
| Strongly agree | 43 (39.1%) |
| I can see the potential value of telepsychiatry for my work |  |
| Neither agree nor disagree | 7 (6.4%) |
| Agree | 38 (34.9%) |
| Strongly agree | 64 (58.7%) |
| There are key people who drive telepsychiatry forward and get others involved |  |
| Disagree | 6 (8.1%) |
| Neither agree nor disagree | 37 (50.0%) |
| Agree | 19 (25.7%) |
| Strongly agree | 12 (16.2%) |
| I believe that participating in telepsychiatry is a legitimate part of my role |  |
| Strongly disagree | 1 (1.0%) |
| Neither agree nor disagree | 4 (3.9%) |
| Agree | 31 (30.1%) |
| Strongly agree | 67 (65.0%) |
| I’m open to working with colleagues in new ways to use telepsychiatry |  |
| Strongly disagree | 1 (1.0%) |
| Disagree | 3 (3.1%) |
| Neither agree nor disagree | 11 (11.3%) |
| Agree | 39 (40.2%) |
| Strongly agree | 43 (44.3%) |
| I will continue to support telepsychiatry |  |
| Disagree | 1 (0.9%) |
| Neither agree nor disagree | 4 (3.7%) |
| Agree | 34 (31.5%) |
| Strongly agree | 69 (63.9%) |
| I can easily integrate telepsychiatry into my existing work |  |
| Disagree | 1 (0.9%) |
| Neither agree nor disagree | 4 (3.8%) |
| Agree | 39 (36.8%) |
| Strongly agree | 62 (58.5%) |
| Telepsychiatry disrupts working relationships |  |
| Strongly disagree | 27 (26.2%) |
| Disagree | 40 (38.8%) |
| Neither agree nor disagree | 22 (21.4%) |
| Agree | 9 (8.7%) |
| Strongly agree | 5 (4.9%) |
| I have confidence in other people’s ability to use telepsychiatry |  |
| Strongly disagree | 1 (1.0%) |
| Disagree | 12 (11.9%) |
| Neither agree nor disagree | 31 (30.7%) |
| Agree | 47 (46.5%) |
| Strongly agree | 10 (9.9%) |
| Work is assigned to those with skills appropriate to telepsychiatry |  |
| Strongly disagree | 2 (2.9%) |
| Disagree | 10 (14.5%) |
| Neither agree nor disagree | 27 (39.1%) |
| Agree | 22 (31.9%) |
| Strongly agree | 8 (11.6%) |
| Sufficient training is provided to enable staff to implement telepsychiatry |  |
| Strongly disagree | 4 (5.3%) |
| Disagree | 17 (22.7%) |
| Neither agree nor disagree | 21 (28.0%) |
| Agree | 21 (28.0%) |
| Strongly agree | 12 (16.0%) |
| Sufficient resources are available to support telepsychiatry |  |
| Strongly disagree | 3 (3.3%) |
| Disagree | 16 (17.6%) |
| Neither agree nor disagree | 22 (24.2%) |
| Agree | 33 (36.3%) |
| Strongly agree | 17 (18.7%) |
| Management adequately supports telepsychiatry |  |
| Strongly disagree | 2 (3.4%) |
| Disagree | 6 (10.3%) |
| Neither agree nor disagree | 9 (15.5%) |
| Agree | 23 (39.7%) |
| Strongly agree | 18 (31.0%) |
| I am aware of reports about the effects of telepsychiatry |  |
| Strongly disagree | 2 (2.1%) |
| Disagree | 11 (11.6%) |
| Neither agree nor disagree | 22 (23.2%) |
| Agree | 50 (52.6%) |
| Strongly agree | 10 (10.5%) |
| The staff agree that telepsychiatry is worthwhile |  |
| Strongly disagree | 1 (1.4%) |
| Disagree | 1 (1.4%) |
| Neither agree nor disagree | 8 (11.1%) |
| Agree | 31 (43.1%) |
| Strongly agree | 31 (43.1%) |
| I value the effects that telepsychiatry has had on my work |  |
| Disagree | 3 (2.9%) |
| Neither agree nor disagree | 11 (10.7%) |
| Agree | 42 (40.8%) |
| Strongly agree | 47 (45.6%) |
| Feedback about telepsychiatry can be used to improve it in the future |  |
| Disagree | 2 (1.9%) |
| Neither agree nor disagree | 9 (8.7%) |
| Agree | 49 (47.1%) |
| Strongly agree | 44 (42.3%) |
| I can modify how I work with telepsychiatry |  |
| Neither agree nor disagree | 15 (14.6%) |
| Agree | 55 (53.4%) |
| Strongly agree | 33 (32.0%) |

**Supplementary Table 2**

Supplementary Table 2 Comparisons of dichotomous NoMAD responses between respondents with high and low telepsychiatry clinical load

| Item | Total | Clinical load | | p-value |
| --- | --- | --- | --- | --- |
|  |  | Low, n (%) | High, n (%) |  |
| **Coherence** |  |  |  |  |
| I can see how telepsychiatry differs from usual ways of working |  |  |  |  |
| Non-affirmative | 23 (20.9%) | 8 (11.8%) | 15 (35.7%) | 0.003* |
| Affirmative | 87 (79.1%) | 60 (88.2%) | 27 (64.3%) |  |
| Staff in this organisation have a shared understanding of the purpose of telepsychiatry |  |  |  |  |
| Non-affirmative | 40 (36.4%) | 29 (42.6%) | 11 (26.2%) | 0.081 |
| Affirmative | 70 (63.6%) | 39 (57.4%) | 31 (73.8%) |  |
| I understand how telepsychiatry affects the nature of my own work |  |  |  |  |
| Non-affirmative | 8 (7.3%) | 4 (5.9%) | 4 (9.5%) | 0.475 |
| Affirmative | 102 (92.7%) | 64 (94.1%) | 38 (90.5%) |  |
| I can see the potential value of telepsychiatry for my work |  |  |  |  |
| Non-affirmative | 8 (7.3%) | 7 (10.3%) | 1 (2.4%) | 0.121 |
| Affirmative | 102 (92.7%) | 61 (89.7%) | 41 (97.6%) |  |
| **Cognitive participation** |  |  |  |  |
| There are key people who drive telepsychiatry forward and get others involved |  |  |  |  |
| Non-affirmative | 79 (71.8%) | 55 (80.9%) | 24 (57.1%) | 0.007* |
| Affirmative | 31 (28.2%) | 13 (19.1%) | 18 (42.9%) |  |
| I believe that participating in telepsychiatry is a legitimate part of my role |  |  |  |  |
| Non-affirmative | 12 (10.9%) | 12 (17.6%) | 0 (0.0%) | 0.004* |
| Affirmative | 98 (89.1%) | 56 (82.4%) | 42 (100.0%) |  |
| I’m open to working with colleagues in new ways to use telepsychiatry |  |  |  |  |
| Non-affirmative | 28 (25.5%) | 23 (33.8%) | 5 (11.9%) | 0.010* |
| Affirmative | 82 (74.5%) | 45 (66.2%) | 37 (88.1%) |  |
| I will continue to support telepsychiatry |  |  |  |  |
| Non-affirmative | 7 (6.4%) | 7 (10.3%) | 0 (0.0%) | 0.032* |
| Affirmative | 103 (93.6%) | 61 (89.7%) | 42 (100.0%) |  |
| **Collective action** |  |  |  |  |
| I can easily integrate telepsychiatry into my existing work |  |  |  |  |
| Non-affirmative | 9 (8.2%) | 8 (11.8%) | 1 (2.4%) | 0.081 |
| Affirmative | 101 (91.8%) | 60 (88.2%) | 41 (97.6%) |  |
| Telepsychiatry disrupts working relationships |  |  |  |  |
| Non-affirmative | 67 (60.9%) | 33 (48.5%) | 34 (81.0%) | <0.001* |
| Affirmative | 43 (39.1%) | 35 (51.5%) | 8 (19.0%) |  |
| I have confidence in other people's ability to use telepsychiatry |  |  |  |  |
| Non-affirmative | 53 (48.2%) | 38 (55.9%) | 15 (35.7%) | 0.040* |
| Affirmative | 57 (51.8%) | 30 (44.1%) | 27 (64.3%) |  |
| Work is assigned to those with skills appropriate to telepsychiatry |  |  |  |  |
| Non-affirmative | 80 (72.7%) | 56 (82.4%) | 24 (57.1%) | 0.004* |
| Affirmative | 30 (27.3%) | 12 (17.6%) | 18 (42.9%) |  |
| Sufficient training is provided to enable staff to implement telepsychiatry |  |  |  |  |
| Non-affirmative | 77 (70.0%) | 58 (85.3%) | 19 (45.2%) | <0.001* |
| Affirmative | 33 (30.0%) | 10 (14.7%) | 23 (54.8%) |  |
| Sufficient resources are available to support telepsychiatry |  |  |  |  |
| Non-affirmative | 60 (54.5%) | 48 (70.6%) | 12 (28.6%) | <0.001* |
| Affirmative | 50 (45.5%) | 20 (29.4%) | 30 (71.4%) |  |
| Management adequately supports telepsychiatry |  |  |  |  |
| Non-affirmative | 69 (62.7%) | 48 (70.6%) | 21 (50.0%) | 0.030* |
| Affirmative | 41 (37.3%) | 20 (29.4%) | 21 (50.0%) |  |
| **Reflexive monitoring** |  |  |  |  |
| I am aware of reports about the effects of telepsychiatry |  |  |  |  |
| Non-affirmative | 50 (45.5%) | 36 (52.9%) | 14 (33.3%) | 0.045* |
| Affirmative | 60 (54.5%) | 32 (47.1%) | 28 (66.7%) |  |
| The staff agree that telepsychiatry is worthwhile |  |  |  |  |
| Non-affirmative | 48 (43.6%) | 32 (47.1%) | 16 (38.1%) | 0.357 |
| Affirmative | 62 (56.4%) | 36 (52.9%) | 26 (61.9%) |  |
| I value the effects that telepsychiatry has had on my work |  |  |  |  |
| Non-affirmative | 21 (19.1%) | 18 (26.5%) | 3 (7.1%) | 0.012* |
| Affirmative | 89 (80.9%) | 50 (73.5%) | 39 (92.9%) |  |
| Feedback about telepsychiatry can be used to improve it in the future |  |  |  |  |
| Non-affirmative | 17 (15.5%) | 13 (19.1%) | 4 (9.5%) | 0.176 |
| Affirmative | 93 (84.5%) | 55 (80.9%) | 38 (90.5%) |  |
| I can modify how I work with telepsychiatry |  |  |  |  |
| Non-affirmative | 22 (20.0%) | 13 (19.1%) | 9 (21.4%) | 0.768 |
| Affirmative | 88 (80.0%) | 55 (80.9%) | 33 (78.6%) |  |
| *Statistically significant |  |  |  |  |

**Supplementary Table 3**

Supplementary Table 3 Characteristics of the participants in semi-structured interviews.

| Sex | Seniority | Psychiatry Specialty | State | Full time private | Fully telehealth |
| --- | --- | --- | --- | --- | --- |
| Male | Mid-career | General | WA | Yes | Yes |
| Female | Senior | General | SA | No | No |
| Female | Mid-career | Perinatal | VIC | Yes | No |
| Male | Junior | General, old age | ACT | Yes | No |
| Male | Senior | General, anxiety disorders, CBT | NSW | Yes | No |
| Male | Senior | General | ACT | Yes | No |
| Male | Senior | General, forensic | NSW | No | No |
| Male | Junior | General, veteran | ACT | Yes | No |
| Male | Junior | General | VIC | No | No |
| Male | Senior | General, adult ADHD | QLD, TAS, SA | Yes | Yes |
| Female | Senior | Child and adolescent | NSW | No | No |
| Female | Mid-career | General | SA | Yes | No |
| Male | Senior | General, child and adolescent | NSW | Yes | Yes |

**Supplementary material: NoMAD questionnaire**

Note: The following questionnaire was adapted from the original NoMAD instrument (Finch et al., 2013). The corresponding author granted permission for the adaptation and use of the instrument. The changes were to “Part A: About yourself”, which was tailored to respondents in psychiatry private practice in Australia, and the substitution of the generic term “the intervention” with “telepsychiatry” throughout the questionnaire.

**Part A: About yourself**

1. Age (years): ______________
2. Gender

- Male
- Female
- I prefer the term: ______________
- I prefer not to say

1. Country of birth

- Australia
- Overseas

1. Work experience in private practice (years): ______________
2. Private practice hours

- Full-time
- Part-time

1. Type of practice

- Solo practice
- Group practice
- Others, please specify: ___________________

1. Private practice area

- Metropolitan
- Non-metropolitan
- Both metropolitan and non-metropolitan

1. Private practice location (tick all that apply)

- ACT
- NSW
- NT
- QLD
- SA
- TAS
- VIC
- WA

1. How long have you practised telepsychiatry?

- Less than 1 year
- ≥ 1 to < 3 years
- ≥ 3 to < 10 years
- ≥ 10 years

1. How often do you practise telepsychiatry?

- Daily
- At least once a week
- At least once a month
- At least once in 3 months
- Less than once in 3 months

1. What is the proportion of your patients seen via telepsychiatry?

- < 5%
- 5% to < 10%
- 10% to <20%
- 20% to < 50%
- ≥ 50%

**Part B: General questions about telepsychiatry**

When you use telepsychiatry, how familiar does it feel?

Still feels very new Feels completely familiar

<------------------------------------------------------>

0 1 2 3 4 5 6 7 8 9 10

Do you feel telepsychiatry is currently a normal part of your work?

Not at all somewhat Completely

<------------------------------------------------------>

0 1 2 3 4 5 6 7 8 9 10

Do you feel telepsychiatry will become a normal part of your work?

Not at all somewhat Completely

<------------------------------------------------------>

0 1 2 3 4 5 6 7 8 9 10

**Part C: Detailed questions about telepsychiatry**

Section 1

This section contains statements on how people in your organisation make sense of what needs to be done with telepsychiatry.

For each statement, please select an answer that best suits your experience using Option A. If the statement is not relevant to you, please select an answer from Option B.

1. I can see how telepsychiatry differs from usual ways of working.

*Option A*

- Strongly agree
- Agree
- Neither agree nor disagree
- Disagree
- Strongly disagree

*Option B*

- Not relevant to my role
- Not relevant at this stage
- Not relevant to the intervention

1. Staff in this organisation have a shared understanding of the purpose of telepsychiatry.

*Option A*

- Strongly agree
- Agree
- Neither agree nor disagree
- Disagree
- Strongly disagree

*Option B*

- Not relevant to my role
- Not relevant at this stage
- Not relevant to the intervention

1. I understand how telepsychiatry affects the nature of my own work.

*Option A*

- Strongly agree
- Agree
- Neither agree nor disagree
- Disagree
- Strongly disagree

*Option B*

- Not relevant to my role
- Not relevant at this stage
- Not relevant to the intervention

1. I can see the potential value of telepsychiatry for my work.

*Option A*

- Strongly agree
- Agree
- Neither agree nor disagree
- Disagree
- Strongly disagree

*Option B*

- Not relevant to my role
- Not relevant at this stage
- Not relevant to the intervention

Section 2

This section contains statements on how relationships with others in your organisation influence the outcomes of telepsychiatry.

For each statement, please select an answer that best suits your experience using Option A. If the statement is not relevant to you, please select an answer from Option B.

1. There are key people who drive telepsychiatry forward and get others involved.

*Option A*

- Strongly agree
- Agree
- Neither agree nor disagree
- Disagree
- Strongly disagree

*Option B*

- Not relevant to my role
- Not relevant at this stage
- Not relevant to the intervention

1. I believe that participating in telepsychiatry is a legitimate part of my role.

*Option A*

- Strongly agree
- Agree
- Neither agree nor disagree
- Disagree
- Strongly disagree

*Option B*

- Not relevant to my role
- Not relevant at this stage
- Not relevant to the intervention

1. I’m open to working with colleagues in new ways to use telepsychiatry.

*Option A*

- Strongly agree
- Agree
- Neither agree nor disagree
- Disagree
- Strongly disagree

*Option B*

- Not relevant to my role
- Not relevant at this stage
- Not relevant to the intervention

1. I will continue to support telepsychiatry.

*Option A*

- Strongly agree
- Agree
- Neither agree nor disagree
- Disagree
- Strongly disagree

*Option B*

- Not relevant to my role
- Not relevant at this stage
- Not relevant to the intervention

Section 3

This section contains statements on how people in your organisation work together to make telepsychiatry practice work.

For each statement, please select an answer that best suits your experience using Option A. If the statement is not relevant to you, please select an answer from Option B.

1. I can easily integrate telepsychiatry into my existing work.

*Option A*

- Strongly agree
- Agree
- Neither agree nor disagree
- Disagree
- Strongly disagree

*Option B*

- Not relevant to my role
- Not relevant at this stage
- Not relevant to the intervention

1. Telepsychiatry disrupts working relationships.

*Option A*

- Strongly agree
- Agree
- Neither agree nor disagree
- Disagree
- Strongly disagree

*Option B*

- Not relevant to my role
- Not relevant at this stage
- Not relevant to the intervention

1. I have confidence in other people’s ability to use telepsychiatry.

*Option A*

- Strongly agree
- Agree
- Neither agree nor disagree
- Disagree
- Strongly disagree

*Option B*

- Not relevant to my role
- Not relevant at this stage
- Not relevant to the intervention

1. Work is assigned to those with skills appropriate to telepsychiatry.

*Option A*

- Strongly agree
- Agree
- Neither agree nor disagree
- Disagree
- Strongly disagree

*Option B*

- Not relevant to my role
- Not relevant at this stage
- Not relevant to the intervention

1. Sufficient training is provided to enable staff to implement telepsychiatry.

*Option A*

- Strongly agree
- Agree
- Neither agree nor disagree
- Disagree
- Strongly disagree

*Option B*

- Not relevant to my role
- Not relevant at this stage
- Not relevant to the intervention

1. Sufficient resources are available to support telepsychiatry.

*Option A*

- Strongly agree
- Agree
- Neither agree nor disagree
- Disagree
- Strongly disagree

*Option B*

- Not relevant to my role
- Not relevant at this stage
- Not relevant to the intervention

1. Management adequately supports telepsychiatry.

*Option A*

- Strongly agree
- Agree
- Neither agree nor disagree
- Disagree
- Strongly disagree

*Option B*

- Not relevant to my role
- Not relevant at this stage
- Not relevant to the intervention

Section 4

This section contains statements on how people in your organisation assess the impact of telepsychiatry.

For each statement, please select an answer that best suits your experience using Option A. If the statement is not relevant to you, please select an answer from Option B.

1. I am aware of reports about the effects of telepsychiatry.

*Option A*

- Strongly agree
- Agree
- Neither agree nor disagree
- Disagree
- Strongly disagree

*Option B*

- Not relevant to my role
- Not relevant at this stage
- Not relevant to the intervention

1. The staff agree that telepsychiatry is worthwhile.

*Option A*

- Strongly agree
- Agree
- Neither agree nor disagree
- Disagree
- Strongly disagree

*Option B*

- Not relevant to my role
- Not relevant at this stage
- Not relevant to the intervention

1. I value the effects that telepsychiatry has had on my work.

*Option A*

- Strongly agree
- Agree
- Neither agree nor disagree
- Disagree
- Strongly disagree

*Option B*

- Not relevant to my role
- Not relevant at this stage
- Not relevant to the intervention

1. Feedback about telepsychiatry can be used to improve it in the future.

*Option A*

- Strongly agree
- Agree
- Neither agree nor disagree
- Disagree
- Strongly disagree

*Option B*

- Not relevant to my role
- Not relevant at this stage
- Not relevant to the intervention

1. I can modify how I work with telepsychiatry.

*Option A*

- Strongly agree
- Agree
- Neither agree nor disagree
- Disagree
- Strongly disagree

*Option B*

- Not relevant to my role
- Not relevant at this stage
- Not relevant to the intervention

Thank you for completing our survey.

**Supplementary material: Topic guide for semi-structured interviews**

Note: This is the topic guide used during the online semi-structured interviews with private practice psychiatrists.

**Introduction**

- Introduce myself and my research role.
- Make sure the information sheet is read and the consent form is signed.
- Ask the participant to briefly introduce themselves:
  - How long have they been doing private practice.
  - Describe their private practice.

**Opening questions**

- Please describe your first encounter with telepsychiatry
- What kind of experience do you have in using telepsychiatry?
- How do you currently use telepsychiatry in your practice?

**Normalisation Process Theory-based questions**

Coherence type questions

- How do you understand the purpose of telepsychiatry?
- Where do you think telepsychiatry should fit into your routine clinical practice?
- From your perspective, how is telepsychiatry different from face-to-face consultation?
- What is your experience with telephone and video telepsychiatry? How are they different from each other (in terms of preferences, benefits, etc.)?
- If relevant, how do colleagues at your practice use telepsychiatry? Do you think they understand the purpose of telepsychiatry in a similar way?
- What potential value do you think telepsychiatry provides? Its benefits and importance?

Cognitive Participation type questions

- Is there any leader or key person who drives the use of telepsychiatry at your practice? What do they do?
- How did your practice plan and prepare for the use of telepsychiatry?
- How do staff at your practice interact with/ use telepsychiatry?
- What do you think may help staff better engage with telepsychiatry?
- Are you keen to support the continued use of telepsychiatry? What are the reasons?

Collection Action type questions

- How well does telepsychiatry becomes a part of your routine clinical practice?
- How does telepsychiatry help/hinder you from working with others?
- Please share any issues you and your staff encounter in running telepsychiatry in terms of:
  - Time and resources
  - Skills and training
  - Management and support, etc.

Reflexive monitoring type questions

- What do you think of the usefulness of telepsychiatry?
- Are you aware of reports of telepsychiatry’s effectiveness, peer-reviewed or anecdotal?
- How does telepsychiatry affect the clinical management of your patients?
- To what extent do you measure or appraise the outcomes of telepsychiatry? Do you collect feedback about its use?
- How does telepsychiatry affect your work quality and work-life balance?
- Do you think telepsychiatry is worthwhile? Please explain.
- How can your current telepsychiatry practice be improved? Have you tried any improvement strategies?

Thank the participant for their time and effort.

**Supplementary material: Codebook**

Note: This is the codebook used for the coding process of the thematic analysis of semi-structured interview content.

| Name | Description |
| --- | --- |
| Cognitive participation | Participation that promotes or inhibits users' enrolment and legitimisation of a practice (Investment of commitment). #How private practice psychiatrists commit themselves to telepsychiatry. |
| Activation | Private practice psychiatrists' commitment to support telepsychiatry, reflected in planning and preparation to set up telepsychiatry services. |
| Enrolment | Collaboration with colleagues in new ways to use telepsychiatry among private practice psychiatrists and staff. |
| Initiation | Key people driving telepsychiatry forward and get others involved among private practice psychiatrists. |
| Legitimation | Seeing telepsychiatry as a legitimate part of the role of private practice psychiatrists. |
| Coherence | Sense-making that promotes or inhibits the coherence of a practice to its users (Investment of meaning). #How private practice psychiatrists make sense of telepsychiatry. |
| Communal specification | How the fraternity of private practice psychiatrists share the understanding of the purpose of telepsychiatry. |
| Differentiation | How telepsychiatry is different from routine (face-to-face) practice. |
| Individual specification | Personal understanding of how telepsychiatry affects the nature of one's private pra ctice. |
| Internalization | Appreciation of the potential value of telepsychiatry in one's private practice. |
| Collective action | Activity that promotes or inhibits the enacting of a practice by its users (investment of efforts). # Efforts taken by private practice psychiatrists to implement telepsychiatry. |
| Contextual integration | Capabilities that support intervention components through resource allocation and mobilization and that link them to their contexts of action. #Sufficient resources (infornational, material, etc.) and managerial/administrative support for telepsychiatry among private practice psychiatrists. |
| Interactional workability | Capabilities that enable participants in an implementation process to operationalize intervention components in practice. #How easy is telepsychiatry integrated into existing work by private practice psychiatrists. |
| Relational integration | Capabilities that promote knowledge about intervention components within networks of participants in an implementation process and which mediate trust and confidence. #Confidence in others' ability to use telepsychiatry and how telepsychiatry affects working relationships in private practice. |
| Skill-set workability | Capabilities that equip participants in an intervention process to perform the work associated with intervention components and which are distributed in a division of labour. #Appropriate skills and sufficient training for private practice psychiatrists to carry out telepsychiatry / ease of use i.e. minimum skill requirements |
| Reflexive monitoring | Practices that promote or inhibits users' comprehension of the effects of a practice. (Investment in appraisal). #How private practice psychiatrists appraise the effects of telepsychiatry. |
| Communal appraisal | Agreement among private practice psychiatrists that telepsychiatry is worthwhile. |
| Individual appraisal | Private practice psychiatrists' appreciation of the effects of telepsychiatry on their own practice. |
| Reconfiguration | Improvement and modification of telepsychiatry by private practice psychiatrists based on feedback. |
| Systemization | Private practice psychiatrists' awareness of the evidence base of telepsychiatry. |
